# Refractory esophageal–mediastinal fistula successfully treated with endoluminal vacuum therapy and enteral nutrition using a double-lumen elemental diet tube: a case report

**DOI:** 10.1186/s44215-023-00114-6

**Published:** 2023-11-23

**Authors:** Shizuka Yoshidome, Ken Sasaki, Hideyuki Fumoto, Yusuke Tsuruda, Masataka Shimonosono, Yasuto Uchikado, Daisuke Matsushita, Takaaki Arigami, Kenji Baba, Hiroshi Kurahara, Takao Ohtsuka

**Affiliations:** 1 Department of Digestive Surgery, Breast and Thyroid Surgery, Graduate School of Medical and Dental Sciences, Kagoshima University, 8-35-1 Sakuragaoka, Kagoshima-Shi, Kagoshima, 890-8520 Japan; 2 Department of Cardiovascular Surgery, Ohsumi Kanoya Hospital, 6081-1 Shinkawa-Machi, Kanoya-Shi, Kagoshima, 893-0015 Japan

**Keywords:** Esophageal–mediastinal fistula, Aortic–esophageal fistula, Endoluminal vacuum therapy, Enteral feeding

## Abstract

**Background:**

Aortic–esophageal fistula (AEF) after thoracic endovascular aortic repair (TEVAR) has a high fatality rate and is difficult to treat. Endoluminal vacuum therapy (EVT) has recently appeared and proven to be a useful method for anastomotic leakage.

**Case presentation:**

A 76-year-old man underwent aortic arch replacement for a stent graft infection after TEVAR. Persistent mediastinitis and pyothorax were observed after aortic arch replacement, and further examination revealed an esophageal–mediastinal fistula (EMF). Over-the-scope clip (OTSC^®^) closure was performed to treat EMF but achieved no cure. Then, the patient was referred to our hospital. First, we removed the OTSC^®^ that interfered with the treatment using the remOVE System^®^ and started EVT using a double-lumen elemental diet tube (W–EDT^®^). The vacuum sponge was affixed to the vacuum side of W–EDT^®^, and enteral nutrition administered through W–EDT was combined with EVT. EMF was cured 11 days after EVT, and the patient was able to feed himself.

**Conclusion:**

The combination of EVT and enteral nutrition feeding using W–EDT® is a successful novel procedure to treat refractory EMF.

## Background

The occurrence of AEF after TEVAR increases as one of the major complications as promising results of TEVAR for thoracic aneurysms promote its usage. AEF has an incidence of approximately 1.5–4% after TEVAR [1, 2], and it is life-threatening and difficult to treat [3, 4]. Esophagectomy combined with aortic replacement can offer a long-term treatment strategy with higher survival rates in patients who develop AEF after TEVAR [5]. Our patient was initially diagnosed with a stent graft infection and underwent total aortic arch replacement. AEF was diagnosed after aortic arch replacement, which caused refractory esophageal–mediastinal fistula (EMF) formation. EVT has been developed recently and is proven to be a useful method to treat intra-thoracic anastomotic leakages after esophagectomy and closure of defects in the upper gastrointestinal tract in addition to stenting [6, 7].

We report a case of refractory EMF successfully treated using EVT in combination with enteral nutrition using a double-lumen elemental diet tube (W–EDT^®^) (Cardinal Health, Dublin, USA).

## Case presentation

**Fig. 1:**
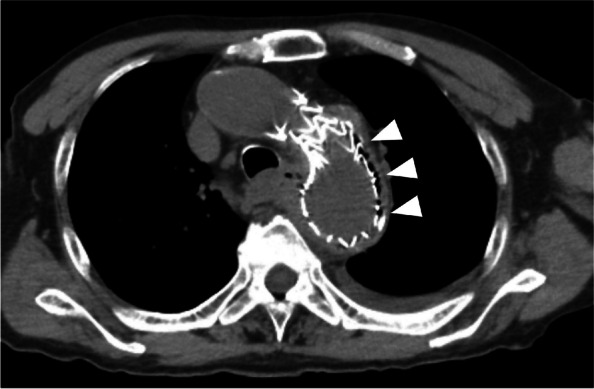
Chest CT findings. Fluid collection and air bubbles were observed around the aortic stent graft (arrowheads)

**Fig. 2:**
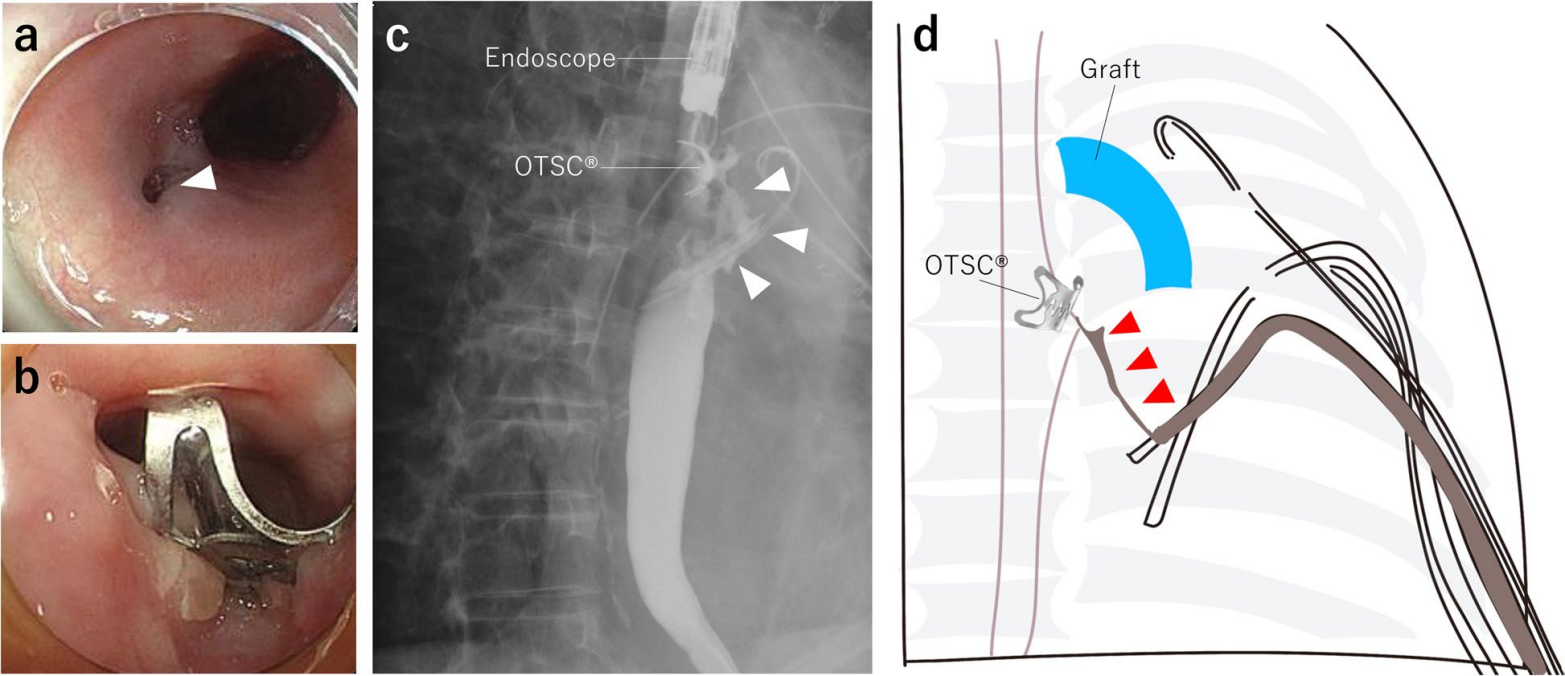
Examination findings and schema of esophageal–mediastinal fistula (EMF). **a** Esophageal–mediastinal fistula (EMF) on the posterior wall of the upper thoracic esophagus (white arrowheads) before suturing with OTSC^®^. **b** EMF after suturing with OTSC^®^. **c** Esophagography showed that EMF (white arrowheads) and OTSC^®^ remained on the oral side of the EMF. **d** Schema of EMF (red arrowheads) upon admission to our hospital

A 76-year-old man presented to the hospital with a chief complaint of fever. He underwent TEVAR to treat a thoracic aortic aneurysm 2 years ago. His computed tomography (CT) revealed fluid collection and air bubbles around his aortic stent graft (Fig. 1), and he was diagnosed with mediastinitis following aortic stent graft infection. He underwent a total aortic arch replacement with omentoplasty for his stent graft infection, and four thoracic drainage tubes were placed in the abscess and his chest cavity. He was observed to have persistent mediastinitis and pyothorax after aortic arch replacement, and further examination revealed that he had an EMF. An endoscopic full-thickness suturing device, the OTSC^®^ system (Ovesco Endoscopy AG, Tübingen, Germany), was used for EMF treatment (Fig. 2a, b), but it was ineffective; thus, the patient was referred to our hospital for refractory EMF treatment.

Physical examination revealed normal vital signs. Laboratory results demonstrated 3.51 mg/L of C-reactive protein. The bacterial culture of pleural discharge revealed the growth of multiple organisms, including *Stenophomonas maltophilia* and *Candida albicans.* Esophagography revealed that EMF led to one of the drainage tubes and was isolated from the replaced aortic arch (Fig. 2c, d). Therefore, we planned to remove OTSC®, which interfered with treatment, and perform EVT for treating EMF.

**Fig. 3:**
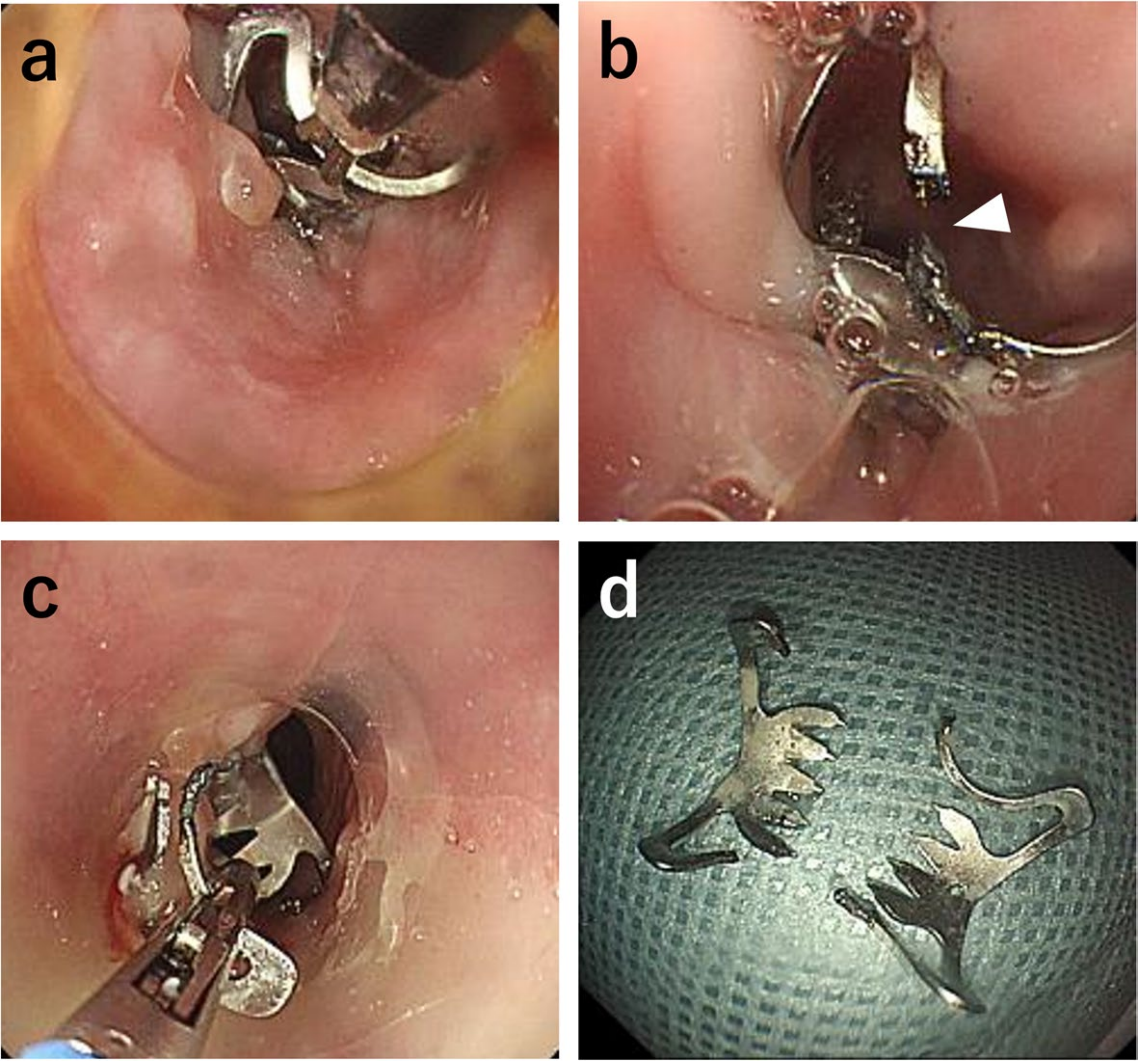
Endoscopic findings. **a** Applying an electrical direct current pulse at two opposing hinges of the clip. **b** The hinge of OTSC^®^ was cauterized and cut into two fragments (white arrowheads). **c** The two fragments were collected with gripping forceps. **d** Collected OTSC^®^ fragments

**Fig. 4:**
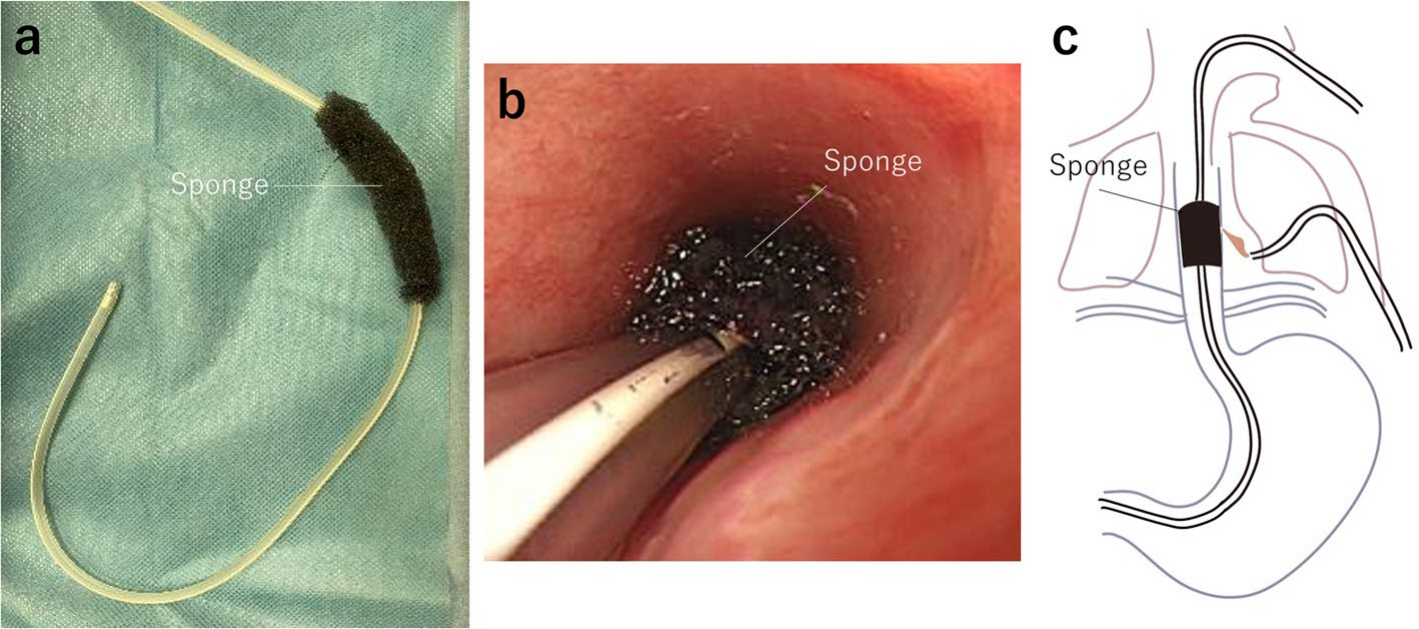
EVT: **a** the wound vacuum sponge was affixed to the drainage site of the double–lumen elemental diet tube (W–EDT^®^). **b** The wound vacuum sponge was located on the esophageal fistula. **c** Schema of EVT and enteral nutrition using W–EDT^®^

**Fig. 5:**
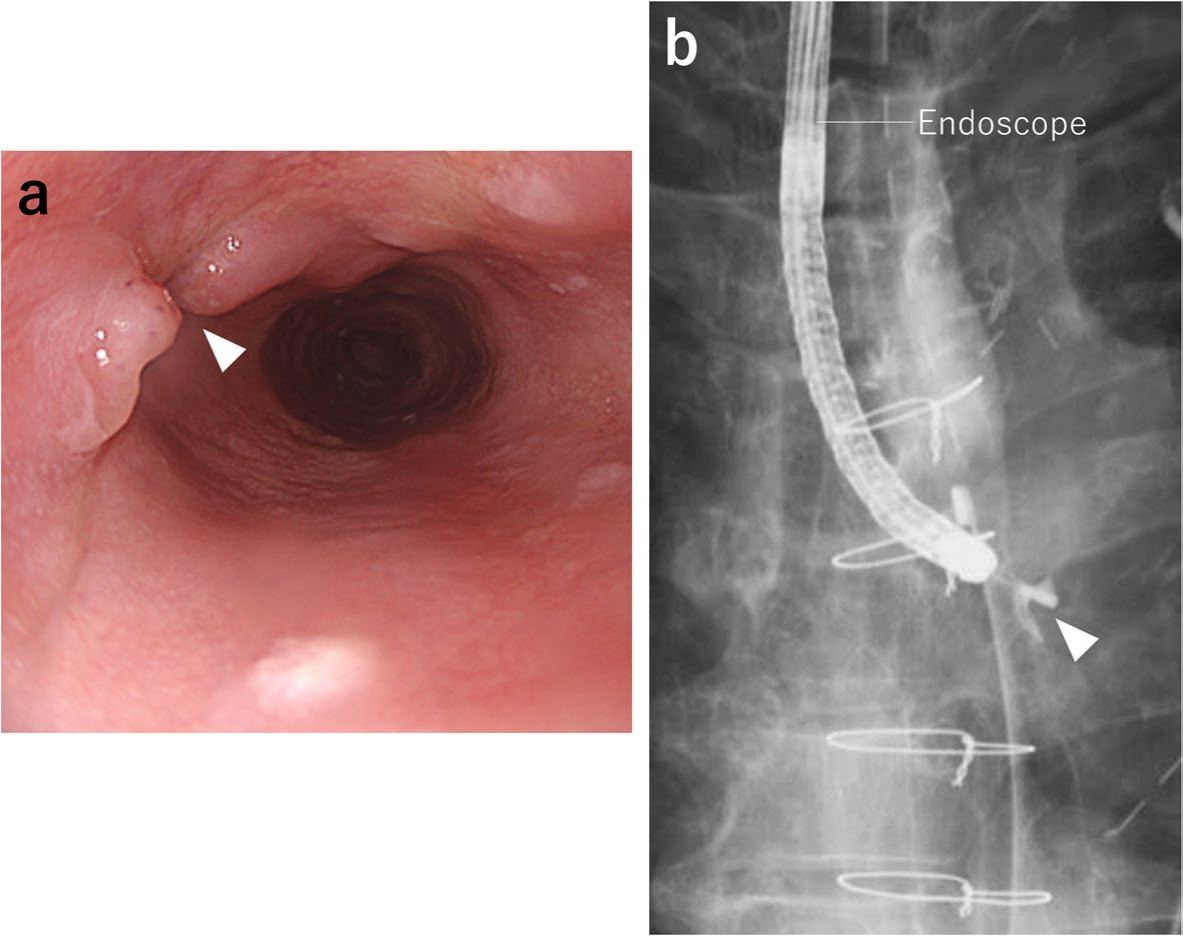
After-treatment findings. **a** Endoscopy confirmed the closure of esophageal–mediastinal fistula (EMF; white arrowhead). **b** Esophageal fistula disappeared on esophagography

First, OTSC^®^ was removed on day 3 of admission using the remOVE System^®^ (Ovesco Endoscopy AG, Tübingen, Germany), which is a medical device for endoscopic OTSC^®^ removal. The standard removal procedure entails fragmenting the clip by applying a direct current pulse at two opposing clip hinges (Fig. 3a, b). Two fragments were collected with gripping forceps through an endoscopic grasper (Fig. 3c, d). Next, the EVT device was inserted into the esophagus. The wound vacuum sponge was affixed to the drainage site of W–EDT^®^ (Fig. 4a) and is located on the EMF (Fig. 4b), and a feeding site of W–EDT^®^ was located in the duodenum (Fig. 4c). A drainage tube was connected to an electric low-pressure suction system, MERA SUCUUM 009^®^ (Senko Medical Instrument, Tokyo, Japan). Thus, continuous vacuum drainage (10 cm H_2_O) and enteral nutrition administration were simultaneously performed. EMF closure was confirmed by endoscopy (Fig. 5a) and esophagography (Fig. 5b) after 11 days of EVT, and the patient started oral intake. The patient was transferred to a referral hospital 23 days after admission, and he was discharged without EMF recurrence 42 days after the transfer.

## Discussion and conclusions

Our patient presented with only a fever although a high incidence of hematemesis and chest pain is recognized in AEF cases [8], and CT indicated stent graft infection as the source of the fever. CT rarely depicts the fistula itself although CT has been reported as a useful examination method for AEF diagnosis, and thus, a careful CT interpretation is needed [9]. Martin et al. have reported that esophagoscopy might be the most sensitive and specific modality for diagnosing AEF [10]. Hai et al. mentioned that 24% of AEF cases had to coexist with aortic stent graft infection cases [11]. Accordingly, we should consider the possibility of the presence of AEF when diagnosing a case with stent graft infection regardless of the typical symptoms or examination findings. Hence, our patient avoided esophagectomy, although the current principal treatment strategy for after-TEVAR AEF is esophageal resection followed by aortic reconstruction [3, 12].

The patient was diagnosed with a stent graft infection and underwent total arch replacement and omentoplasty. Persistent mediastinitis and pyothorax were seen postoperatively, and further examination revealed an EMF. Fortunately, the aortic infection was controlled upon hospital admission, and EMF was isolated from the total aortic arch graft. Therefore, our goal was the closure of the refractory EMF. Watanabe et al. demonstrated that both esophagectomy and aortic replacement effectively prolonged the survival of patients with AEF after TEVAR, and they suggested that esophagectomy may be mandatory to achieve a long-term survival rate despite esophagectomy being a high-risk surgery from the results of the questionnaire survey [5]. However, if a replaced aortic graft is completely isolated from EMF, esophagectomy may not always be necessary for the results of this case study.

In general, treatment methods for EMF include OTSC^®^, a self-expanding metal stent (SEMS), and EVT. OTSC^®^ is a simple and effective method with a 90% successful closure rate when the wound edge is cleaned from leaks due to esophageal rupture. However, the closure rate decreases greatly in cases with chronic inflammation or insufficient infection site drainage [13]. This case was referred to our hospital after OTSC without success. If the patient had been treated at our hospital from the beginning, we would have performed EVT and W-EDT instead of OTSC. Because OTSC is an effective method for EMF with a clean wound edge, without chronic inflammation or abscess. In addition, OTSC^®^ is disadvantageous because it is difficult to remove when fistula closure is unsuccessful. Methods, such as endoscopic mucosal resection and argon plasma cauterization, have also been reported as OTSC^®^ removal methods, but all are highly invasive. A systematic review reported that remOVE System^®^ eliminated the OTSC^®^ with an 85–93% success rate, making it the safest and most effective [14]. SEMS has been considered a standard treatment method for refractory EMF, with an 81.1% closure rate [15]. However, stent migration and dislocation are major limitations of SEMS [16]. Conversely, EVT, which has recently become more widely used, has been reported to be effective. Laukoetter et al. have reported a 94.2% closure rate of EVT in EMF [7]. Meta-analysis data demonstrated a significantly higher closure rate and a significantly lower incidence rate of major complications in EVT than those in SEMS [17]. A sponge was wrapped around the single-lumen drainage tube, inserted into the leakage cavity or the lumen of the esophagus, and connected through a drainage tube to a continuous negative pressure ranging from 75 to 175 mmHg for EVT methods reported in previous studies. We used 7.5 mmHg of continuous negative pressure and placed a sponge for 11 days on the esophageal fistula. We judged 7.5 mmHg of continuous negative pressure to be sufficient, although the negative pressure we used was extremely lower than that used by other authors, because we placed the sponge in close contact with the EMF and confirmed no air leakage after starting negative pressure, and it may reduce the incidence risk of complications, such as stricture formation [18]. In addition, EVT and enteral nutrition were simultaneously performed using W–EDT^®^ on our patient, which has tip holes for enteral nutrition and side holes for drainage at 40 cm from the tip. An additional transnasal feeding tube is unavailable, and patients sometimes require a percutaneous endoscopic gastrostomy or a jejunostomy when a sponge is placed in the esophageal lumen [7]. EVT with W–EDT^®^ is a minimally invasive and efficient method to perform vacuum therapy while maintaining the nutrition status of patients. Recent reports indicated a new esophageal stent in combination with an EVT sponge allowing EVT with oral intake [19]. Based on this case study and previous reports as described above, we summarize the types of therapeutic procedures and their indications for each type of EMF in Table 1.

**Table 1:** Summary of therapeutic procedures for EMF

Procedure	Success rate (%)	Merits	Demerits	Indication for types of EMF	Ref. No.
OTSC	42.9% (*n* = 108)	Simple	Not suitable for fistula hole larger than 20 mm Difficult to remove when unsuccessful	Wound less than 20 mm Without inflammation and abscesses	[13, 14]
SEMS	81.1% (*n* = 340)	Oral intake available	Stent migration and dislocation	With inflammation but without abscesses	[15, 16]
EVT (Intraluminally or intracavity)	94.2% (*n* = 52)	Low incidence of major complications	Oral intake unavailable	Localized abscesses endoscopically accessible	[7, 17]

*EMF* Esophageal mediastinal fistula, *OTSC* Over the scope clip, *SEMS* Self-expanding metal stent, *EVT* Endoluminal vacuum therapy

In conclusion, the combination of EVT and enteral nutrition using W–EDT is a novel procedure for EMF, which could accelerate the healing of EMF and reduce the time period of hospitalization.

## Data Availability

All data generated or analyzed during this study are included in this published article.

## Abbreviations

TEVAR: Thoracic endovascular aortic repair
AEF: Aortic–esophageal fistula
EMF: Esophageal–mediastinal fistula
SEMS: Self-expanding metal stent
EVT: Endoluminal vacuum therapy
W–EDT: Double-lumen elemental diet tube
CT: Computed tomography
OTSC: Over-the-scope clip
